# Network analysis of attention deficit hyperactivity disorder traits and mental health indicators in Brazilian adults

**DOI:** 10.7717/peerj.21802

**Published:** 2026-11-03

**Authors:** Daniel de Freitas Quintanilha, Diogo Fagundes Pereira, Dimitri Marques Abramov

**Affiliations:** 1 Laboratory of Neuroscience and Mental Health, Arthur Sá Earp Neto University Center, Petrópolis, Rio de Janeiro, Brazil; 2 Laboratory of Neurobiology and Clinical Neurophysiology, Fernandes Figueira Institute, Oswaldo Cruz Foundation, Rio de Janeiro, Rio de Janeiro, Brazil

**Keywords:** ADHD, Traits, Network analysis, General population, Mental health, Screening, Survey, Neurodiversity, Neurodevelopment, Transcultural

## Abstract

Attention-Deficit/Hyperactivity Disorder (ADHD), a neurodevelopmental disorder characterized by behavioral traits such as inattention, hyperactivity, and impulsivity, has been widely associated with mental health impairments. However, the presence of these traits alone does not determine diagnosis, as they must also cause significant functional impairment for the individual to fulfill diagnostic criteria. Therefore, this study aimed at exploring how ADHD traits are associated with mental health indicators in general population adults regardless of whether participants met full diagnostic criteria for the disorder. The Adult ADHD Self-Report Scale (ASRS) was used to screen ADHD traits in an online survey that also addressed mental health indicators such as excessive preoccupation, lack of meaning in life and substance use. Invitations were distributed *via* social media platforms. The final sample consisted of 2,159 Brazilian adult participants. Data were analyzed using network analysis. An undirected network was modeled using the Gaussian Graphical Model based on partial polychoric correlations, and regularization was applied using EBICglasso. The main network analysis was conducted for individual ADHD traits. Additional analyses were performed for the summed inattention and hyperactivity/impulsivity scores, for the total ASRS summed score, and for subgroups of participants exhibiting extreme levels of trait presentation. Results indicate that, in an adult community sample, association between ADHD traits and mental health impairment is predominantly indirect. This indirect association was commonly interposed by younger age and feelings of insecurity. The results were consistent across all analyses. Overall, our results suggest that the presence of ADHD traits alone is not strongly associated with mental health impairment in the general population. We propose that both research and clinical practice may benefit from further exploring and addressing intervening factors—such as insecurity—that could underlie the association between ADHD traits and mental health impairment observed in previous studies.

## Introduction

Attention-Deficit/Hyperactivity Disorder (ADHD) is a neurodevelopmental disorder characterized by a persistent pattern of inattention and/or hyperactivity/impulsivity that interferes with functioning or development. Global prevalence is estimated at 5.9% in children and 2.5% in adults, with a higher prevalence in males (Faraone et al., 2021). According to the Diagnostic and Statistical Manual of Mental Disorders—fifth edition (DSM-5) (American Psychiatric Association, 2022), ADHD is characterized by the presence of certain behavioral traits (inattention, hyperactivity, and impulsivity), which must be associated with clear evidence of interference to the person’s social, academic, or occupational functioning to fulfill diagnosis, among other criteria such as presence in two or more settings and onset prior to 12 years of age.

ADHD is a highly heterogeneous diagnosis, and one of the possible reasons for that is the elevated rate of comorbidities (Gnanavel et al., 2019). Multiple mental health disorders are more common in ADHD individuals than in neurotypical ones, such as substance use, mood, anxiety, and personality disorders (Choi et al., 2022). ADHD individuals have higher prevalence of sleep disorders (Lugo et al., 2020), panic and post-traumatic stress disorders (Adler et al., 2019), and suicidal behaviors (Fitzgerald et al., 2019). On the other hand, individuals with bipolar, borderline personality, and obsessive-compulsive disorder have higher rates of ADHD diagnoses than the general population; such elevated levels of bidirectional comorbidity complicate the definition of ADHD as a singular disorder (Heidbreder, 2015).

Although ADHD is commonly conceptualized as a discrete entity (*i.e.,* a person could only have it or not), there is not sufficient evidence for such a discontinuity; on the contrary, some authors propose ADHD as a “psychophysiological modality” that presents itself as a heterogeneous spectrum which is not limited to pathological states and is present in the general population (Heidbreder, 2015). Being at the extreme of this continuum is not determinant of diagnosis. According to diagnostic criteria, individuals who are highly inattentive, hyperactive, and/or impulsive, but do not display significant impairment because of that, could not be classified as having ADHD, but simply as having a particular cognitive profile. This cognitive profile may hold potentials and vulnerabilities that are different from those of more neurotypical individuals but would not characterize a psychopathology *per se* (Schippers, Greven & Hoogman, 2024).

Since the presence or absence of a single symptom can determine whether an individual meets the threshold for diagnosis, clear distinction between individuals with and without ADHD can be challenging (Silk et al., 2019). Therefore, differential diagnosis in ADHD is of major importance. Both attentional difficulties and psychomotor agitation are common to other disorders, such as major depression and generalized anxiety disorders (American Psychiatric Association, 2022). ADHD traits are influenced by a variety of factors, many of which are correlated with stress (Gbessemehlan et al., 2020) and partially overlap with subjective suffering, like in the relationship between hyperactivity and anxiety, which could complicate assessment specificity (Alarachi et al., 2024).

Until now, no purely biological ADHD diagnosis predictor has been found  (Faraone et al., 2021), but several environmental factors that impact children development have been associated with ADHD, such as childhood maltreatment (Ouyang et al., 2008), abuse, and attachment issues (Richards, 2013), as well as parental low educational attainment, unemployment, and relative income poverty (Keilow, Wu & Obel, 2020). Although its causes are not yet well understood, ADHD has several consequences in the lives of the individuals, both in childhood and in adulthood. Receiving an ADHD diagnosis in adulthood predicts lower labor market integration (Chen et al., 2024). ADHD in adults is also related to unemployment and decreased earnings (Fletcher, 2014).

On the other hand, some evidence suggests that the relationship between ADHD traits and functional impairment is context dependent. For example, some evidence suggests that athletes present more ADHD traits than non-athletes (Ekman, Hiltunen & Gustafsson, 2021). ADHD is also associated with entrepreneurship  (Moore, McIntyre & Lanivich, 2021), especially in the hyperactivity dimension (Sônego et al., 2021). Most individuals report there are positive characteristics associated with ADHD (Mahdi et al., 2017). Creativity is among the most common of those characteristics, but also dynamism, flexibility, and socioaffective abilities (Schippers et al., 2022). Even some characteristics which are related to core symptoms of ADHD might be experienced as positive by high functioning individuals, like being always energized—related to hyperactivity—or having hyperfocus—which could be interpreted as a deficit in attention switching (Sedgwick, Merwood & Asherson, 2019). It has also been found that, even in the general population, ADHD traits are positively correlated with sensitivity, hyperfocus, and flexibility  (Schippers, Greven & Hoogman, 2024).

This evidence of diagnostic overlap, etiological unspecificity, and context dependent functional impact may build a case for ADHD to be seen as a spectrum, rather than a singular entity (Heidbreder, 2015). However, how ADHD related traits are associated with mental health outcomes in the general population is a matter not fully understood. Identifying mental health indicators that are directly associated with ADHD traits in general population adults would allow functional impairment and distress to be predicted, addressed and prevented independently of full diagnostic criteria being met. To fulfill this role, this study aimed at exploring how ADHD traits are associated with mental health indicators in general population adults regardless of whether participants met full diagnostic criteria for the disorder. By treating them separately and as continua, this study sought to advance our understanding of how ADHD traits interact with mental health outcomes.

To analyze our dataset, we chose the approach of network analysis. Network analysis in mental health is a growing field of research, whose focus is to model the complexity of mental phenomena (Boschloo et al., 2015). It stems from the idea that mental phenomena in general, and mental disorders particularly, are emergent properties of the interaction between an array of elements (Borsboom, Cramer & Kalis, 2019). Emergent properties are manifestations that cannot be predicted from the isolated elements of the system because they are derived from the organization of the collectivity of elements  (Cramer et al., 2010).

Some studies have previously applied network analysis to ADHD data. It has been found that hyperactivity/impulsivity symptoms are more densely connected to each other than inattention symptoms in ADHD children  (Silk et al., 2019). In neuropsychological data, it was found that ADHD subjects’ performance in the cognitive tests form a denser network than the performance of neurotypical peers, which indicates that the cognitive abilities they assess are more codependent in ADHD individuals (Guo et al., 2023). In a Brazilian study, a sample of more than 7,000 nonclinical children had their data concerning ADHD and autism spectrum disorder (ASD) traits analyzed. Results indicated that some traits from both conditions are highly correlated, especially hyperactivity traits from ADHD and social communication traits from ASD, possibly having confounding effects on diagnosis (Farhat et al., 2022). Recently, it has also been found that emotional symptoms have bridging effects on inattention and hyperactivity/impulsivity traits (Gauld et al., 2025). In a series of studies encompassing more than 6,000 adults, network analysis reinforced the distinction between ADHD traits and ASD traits, although attention control was found to be a possible transdiagnostic process common to both conditions (Waldren et al., 2024).

Network analysis is a growing paradigm in mental health theory and research (Borsboom, Cramer & Kalis, 2019), and we expect its application to generate novel and meaningful insights about the relationship between mental health and ADHD traits in adults from the general population. We also believe that the analysis of data from a sample of Brazilian individuals would provide new perspectives into ADHD research and contribute to the transcultural replication of mental health research findings.

## Materials & Methods

### ADHD screener

The Adult ADHD Self-Report Scale (ASRS) is an instrument developed in collaboration with the World Health Organization (WHO) for screening ADHD symptoms in adults (Kessler et al., 2005). ASRS is the most widely used tool to support adult ADHD assessment (Choi et al., 2022). Its items were designed to correspond to the symptoms of ADHD listed in the Diagnostic and Statistical Manual of Mental Disorders, fourth edition (DSM-IV), which have been retained in the current fifth edition (American Psychiatric Association, 2022).

The ASRS is divided into two parts: Part A comprises six items identified as the best predictors of positive screening in clinical ratings, outperforming the full version. The first four items refer to inattention symptoms (*e.g.*, “How often do you have trouble wrapping up the final details of a project, once the challenging parts have been done?”), while the other two refer to hyperactivity/impulsivity symptoms (*e.g.*, “How often do you fidget or squirm with your hands or feet when you have to sit down for a long time?”). Part B consists of the remaining twelve items as to cover all ADHD symptoms listed in the DSM-IV. Responses are provided on a five-point scale ranging from 0 (never) to 4 (very often). In this study, we applied Part A items for optimizing both accuracy and parsimony.

Scoring can be made in two ways. At first, the authors proposed dichotomizing each item into absent or present and adopting 4 present symptoms as a cutoff. However, it was later found that summing the responses to all items and adopting a 14-point cutoff for Part A items outperformed the dichotomization scoring method (Kessler et al., 2007). Therefore, the summation method was employed in this study.

### Data collection

This study is a cross-sectional online survey. It was approved by the ethics committee of the Fernandes Figueira Institute in accordance with the Declaration of Helsinki (approval number: 2.473.379). Data collection occurred between February 5th and May 2nd of 2018. Written online informed consent was obtained at the first page of the survey. No information that could identify individual participants was obtained.

Participants were invited to answer a fully online survey on Google Forms titled “Relationships between Behavior, Health, and Working Life”, naïve that it included ASRS Part A. ADHD was not mentioned to prevent sample bias towards individuals interested in it. The survey was in Brazilian Portuguese. No incentive was offered for participation. Sampling was conducted using convenience sampling, with invitations distributed *via* social media platforms through posts in groups and in the profiles of acquaintances of the researchers. Aimed sample size was not pre-determined, but the data collection period was set to last three months.

### Survey description

We collected data on general mental health indicators through twenty items designed for this survey. We aimed to address questions commonly asked in clinical interview assessments. All were answered on a five-point scale (from 0 = never to 4 = very often). Participants also reported information like whether they had attended psychotherapy, some work-related data, and sociodemographic data, including sex, gender, age, income, and educational level.

Those variables are detailed in Table 1. Each variable was assigned a short name as a label to facilitate reference.

**Table 1:** Variables description.

**Label**	**Variable**	**Values**
ASRS 1	Difficulty doing tasks	Likert scale: 0 = Never; 1 = Rarely; 2 = Sometimes; 3 = Often; 4 = Very often
ASRS 2	Disorganization	
ASRS 3	Forgetting appointments	
ASRS 4	Procrastination	
ASRS 5	Psychomotor agitation	
ASRS 6	Restlessness	
age	Age in years	Number of years
income	Monthly income in R$	12 ordinal categories with a R$1,500 interval for each one, with 0 being no income and 11 being income above R$15,000
educ.level	Educational level	Eight ordinal categories for each educational level, with 0 being no formal educational level and 7 being complete graduation
bio.sex	Biological sex	0 = male; 1 = female
work.suf	How do you feel about your job?	0 = it gives me pleasure and joy; 1 = it gives me psychological suffering
prof.chang	Would you change your profession?	0 = no; 1 = yes
therapy	Do you go or have gone to therapy?	0 = no; 1 = yes
1- insecure	Insecure in performing tasks	Likert scale: 0 = Never; 1 = Rarely; 2 = Sometimes; 3 = Often; 4 = Very often
2- crowd	Feeling distress in crowded places	
3- wait	Difficulty waiting in lines	
4- sex.st	Excessive need for sexual stimuli	
5- preoc	Excessive preoccupation	
6- drink	Difficulty in stopping drinking	
7- friend	Ease of making new friends	
8- creat	Finding creative solutions for everyday problems	
9- sub.use	Use of substances apart from alcohol	
10- sub.fq	Frequency of past substance use	
11- sub.comp	Compulsive substance use	
12- meaning	Feeling life has no meaning	
13- emotion	Getting emotional frequently	
14- sl.no.med	Able to sleep without medication	
15- loved	Feeling excessive need to be loved	
16- ch.vio	Experiencing violence in childhood	
17- ch.rej	Experiencing rejection in childhood	
18- sl.df	Difficulty sleeping naturally	
19- pr.comp	Having received parental comprehension	
20- sub.dis	Substance use when in distress	

### Data analysis

We performed network analysis using the R software (v. 4.4.0; R Core Team, 2024) bootnet (v. 1.6) and qgraph (v. 1.9.8) packages. An undirected network was modeled through partial correlations using the Gaussian Graphical Model (GGM) with polychoric correlations, which are adequate for datasets with mixed data (Epskamp, Borsboom & Fried, 2018). With the use of partial correlations, network analysis estimates the correlation between two variables controlling for their correlations with all other variables. For network regularization, Extended Bayesian Information Criterion and Graphical Least Absolute Shrinkage and Selection Operator (EBICglasso) was employed, which eliminates probably spurious correlations, controlling to avoid excessive sparsity (Hevey, 2018). The hyper-parameter was set to 0.5.

Graphical representations are commonly used in network analysis. In these graphs, variables are called nodes, and their links are called edges, which can be weighted (representing magnitude of association) or not (representing merely presence or absence of association). Centrality measures that quantify how much each node influences the network can also be calculated (Hevey, 2018). The following centrality measures were assessed in this study: strength, expected influence, closeness and betweenness.

Strength is the sum of the absolute values of the edges connected to a node, while in expected influence the edges raw values are summed. Therefore, both strength and expected influence showcase the magnitude of a node’s direct links to other nodes. Closeness is the inverse of the sum of the edges between the node and all the other nodes: in other words, closeness represents the node’s association to all other nodes directly or indirectly connected to it. Finally, betweenness indicates the frequency in which the shortest path between two nodes goes through the node in question, meaning that nodes with high betweenness serve as bridges between other nodes.

Confidence intervals (CI) of 95% were obtained through bootstrap with 10,000 resamples to assess edges accuracy. A large confidence interval means that an edge does not hold enough accuracy, since it is unstable in resampling. For the accuracy of the centrality measures, 95% CI obtained through case-dropping bootstrap with 10,000 resamples was employed. Correlation stability (CS) coefficient was calculated, which represents how stable centrality measures are in subset bootstrap samples. A CS coefficient less than 0.25 was considered unacceptable; a coefficient higher than 0.50 was considered acceptable and a coefficient higher than 0.75 was considered ideal (Epskamp, Borsboom & Fried, 2018).

The twenty items related to mental health indicators were developed for use in this study and not derived from an already validated instrument. Therefore, evidence of validity was assessed for these items through Exploratory Factor Analysis (EFA) using the software Factor (12.06.08)  (Lorenzo-Seva & Ferrando, 2006). Following guidelines for best practices in EFA, analysis specifications were: polychoric correlation matrix, optimized parallel analysis with 500 correlation matrices and permutation of raw data for factor retention, robust diagonally weighted least squares (DWLS) for factor extraction, promin for factor rotation, bias-corrected and accelerated (BCa) bootstrap with 500 samples for 95% confidence intervals, and 0.3 as threshold for factor loadings (Rogers, 2022). The same procedures were applied to assess ASRS items’ evidence of validity.

Additional procedures to ensure the validity of the findings were taken in order to assess the effect of redundant items in the results. Redundant items are those that have high intercorrelation and have similar correlation with other items in the network, which indicates that they reflect essentially the same behavior (Christensen, Golino & Silvia, 2020). Redundant items may distort the network topography and bias interpretation. Redundancy analysis was made through the EGAnet package (v. 2.4.1) using the Unique Variable Analysis (UVA) function, employing the weighted topological overlap (wTO) with a cutoff of 0.25 (Christensen, Garrido & Golino, 2023). Each redundant node pair was then reduced to one node representing their mean.

## Results

### Sample description

The total sample size was 2,173 subjects. Fourteen subjects who left one or more ASRS items blank were excluded (mean age = 37.67, biological sex = five males and eight female [1 missing], gender = 12 cisgender and one noncisgender [1 missing], median income level = 1.50, median education level = 6, mean ASRS score = 7.64).

After exclusion, the final sample was made up of 2,159 participants. Table 2 contains sample information regarding socioeconomic data. Gender was not included in further analyses due to high overlap with biological sex (97.96% of participants were cisgender). A total of 482 participants (22.33%) scored 14 or more on the ASRS, classifying as positive screens (141 male, or 21.83% of all male participants; 341 female, or 22.58% of all female participants).

**Table 2:** Sample socioeconomic data.

	***n*(%)**
Biological sex	Female = 1,510 (69.94%)
	Male = 646 (29.92%)
	Blank = 3 (0.14%)
Age (x¯ = 40.33, sd = 12.20)	From 18 to 25 = 231 (10.70%)
	From 26 to 35 = 634 (29.37%)
	From 36 to 45 = 570 (26.40%)
	From 46 to 55 = 381 (17.65%)
	From 56 to 65 = 265 (12.27%)
	From 66 to 78 = 43 (1.99%)
	Blank = 35 (1.62%)
Income in Real (R$) (median = 2)	0 - No income = 342 (15.84%)
	1 - Up to 1,500 = 346 (16.03%)
	2 - From 1,501 to 3,000 = 407 (18.85%)
	3 - From 3,001 to 4,500 = 307 (14.22%)
	4 - From 4,501 to 6,000 = 208 (9.63%)
	5 - From 6,001 to 7,500 = 3 (0.14%)
	6 - From 7,501 to 9,000 = 138 (6.39%)
	7 - From 9,001 to 10,500 = 85 (3.94%)
	8 - From 10,501 to 12,000 = 71 (3.29%)
	9 - From 12,001 to 13,500 = 43 (1.99%)
	10 - From 13,501 to 15,000 = 56 (2.59%)
	11 - More than 15,000 = 124 (5.74%)
	Blank = 29 (1.34%)
Education level (median = 6)	0 - None = 9 (0.42%)
	1 - Middle school = 33 (1.53%)
	2 - Incomplete High School = 39 (1.81%)
	3 - Complete High School = 154 (7.13%)
	4 - Technical Education = 86 (3.98%)
	5 - Incomplete Undergraduation = 321 (14.87%)
	6 - Complete Undergraduation = 552 (25.57%)
	7 - Graduation = 965 (44.70%)

### Evidence of validity

In EFA for mental health items, parallel analysis advised adopting 2 factors (Fig. S1). Goodness of fit indices suggest fair fit of the model (Rogers, 2022): Comparative Fit Index (CFI) = 0.923, Non-Normed Fit Index = 0.903 and Root Mean Square Error of Approximation (RMSEA) = 0.080 (95% CI [0.0755–0.0818]).

Factor loadings (Fig. S2) indicated items 1, 2, 3, 5, 8, 12, 13, 14, 15, 16, 17, 18, and 19 to load into factor 1. Analysis of the content of these items (Table 1) suggests that Factor 1 represents a general indicator of mental health distress, risk factors and functional impairment. Items 6, 9, 10, 11, and 20 were loaded at factor 2. Those are all the items in the survey related to substance use, which seems to be the main content of factor 2. Items 4 (excessive need for sexual stimuli) and 7 (ease of making new friends) did not load into any factor above the 0.3 threshold.

The items of the mental health survey showed fair evidence of validity based on internal structure and are therefore considered adequate for use in network analysis. Exceptions are items 4 and 7. However, EFA does not rule out that these items might have good validity; instead, it indicates that no evidence of validity was found through association with other items. For that reason, items 4 and 7 will be included in further analysis, although special caution is needed in interpreting any result derived directly from them.

In EFA for ASRS items, parallel analysis advised adopting 1 factor (Fig. S3). Goodness of fit indices also suggested fair fit of the model  (Rogers, 2022): Comparative Fit Index (CFI) = 0.962, Non-Normed Fit Index = 0.937 and Root Mean Square Error of Approximation (RMSEA) = 0.076 (95% CI [0.0561–0.0891]). Although items 5 and 6, both the items from the hyperactivity/impulsivity dimension, showed low loading to the factor (Fig. S4), this result suggests that the six items from ASRS part A can be treated as a unidimensional measure with fair fit. However, should more hyperactivity/impulsivity items be added, it is possible they would form a separate factor. Overall, results show adequate evidence of validity for ASRS items based on internal structure.

### Network accuracy measures

Measures of accuracy of the network presented in Fig. 1 indicate that low magnitude edges display low accuracy and should be interpreted with caution. Overall, the results suggest adequate accuracy.

**Figure 1:**
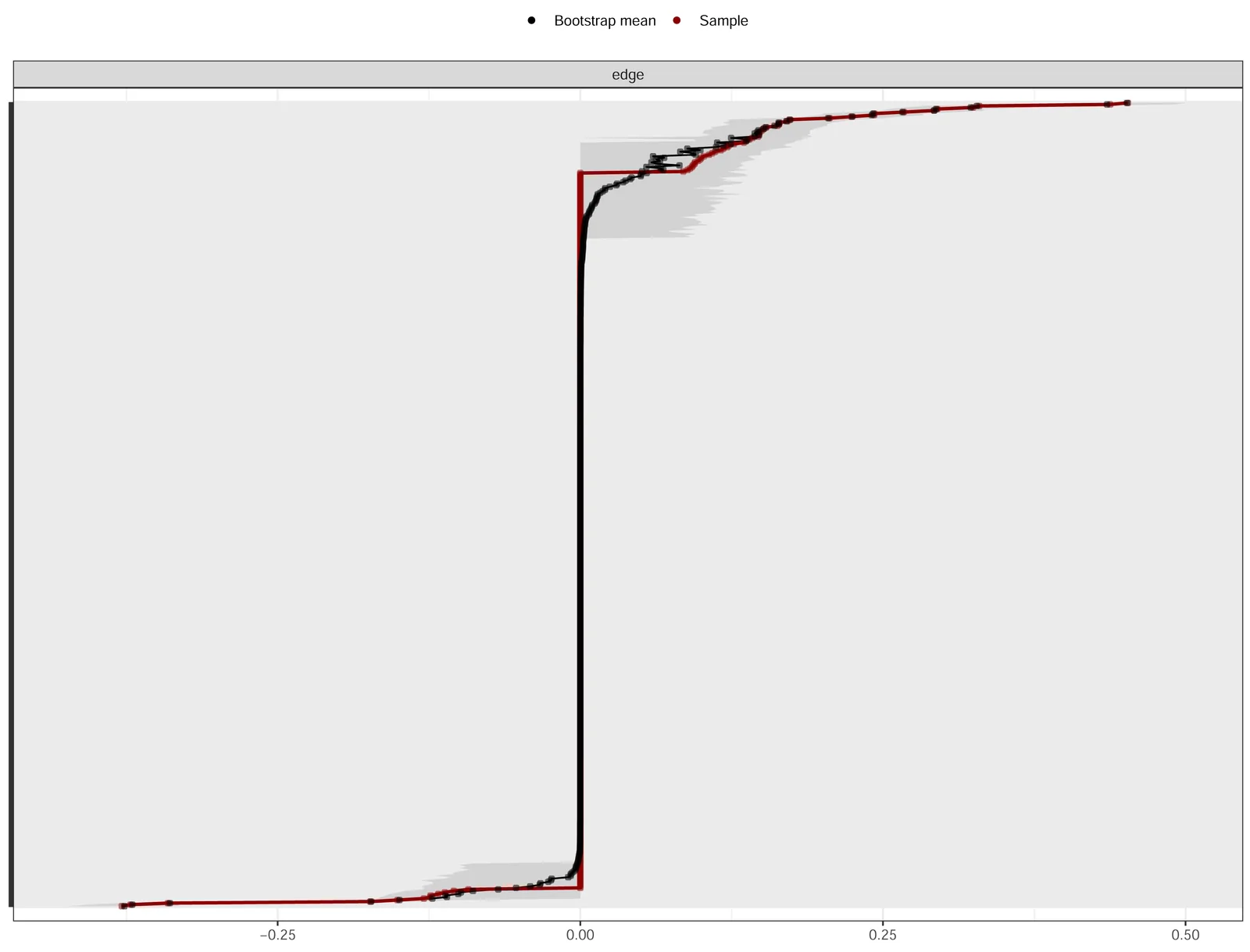
Accuracy of network edges. Red line represents the values of the edges derived directly from the sample, whereas the black line represents the mean values of the edges derived from the bootstrapped samples. Names of the 528 edges are omitted from the *Y* axis in order to enhance readability. The darker gray area represents the 95% confidence interval generated from bootstrap. Negative edges are below, and positive edges are above. Some low magnitude edges confidence intervals contain zero. However, overall results suggest adequate accuracy.

Figure 2 presents accuracy for centrality measures. CS coefficients were 0.516 for strength, 0.750 for expected influence, 0.000 for closeness and 0.050 for betweenness. Therefore, while strength and expected influence had acceptable and ideal accuracy, respectively, closeness and betweenness had inadequate accuracy. This is a common result for psychological networks (Epskamp, Borsboom & Fried, 2018). Thus, expected influence values can be further interpreted, and strength values as well, although with more caution.

**Figure 2:**
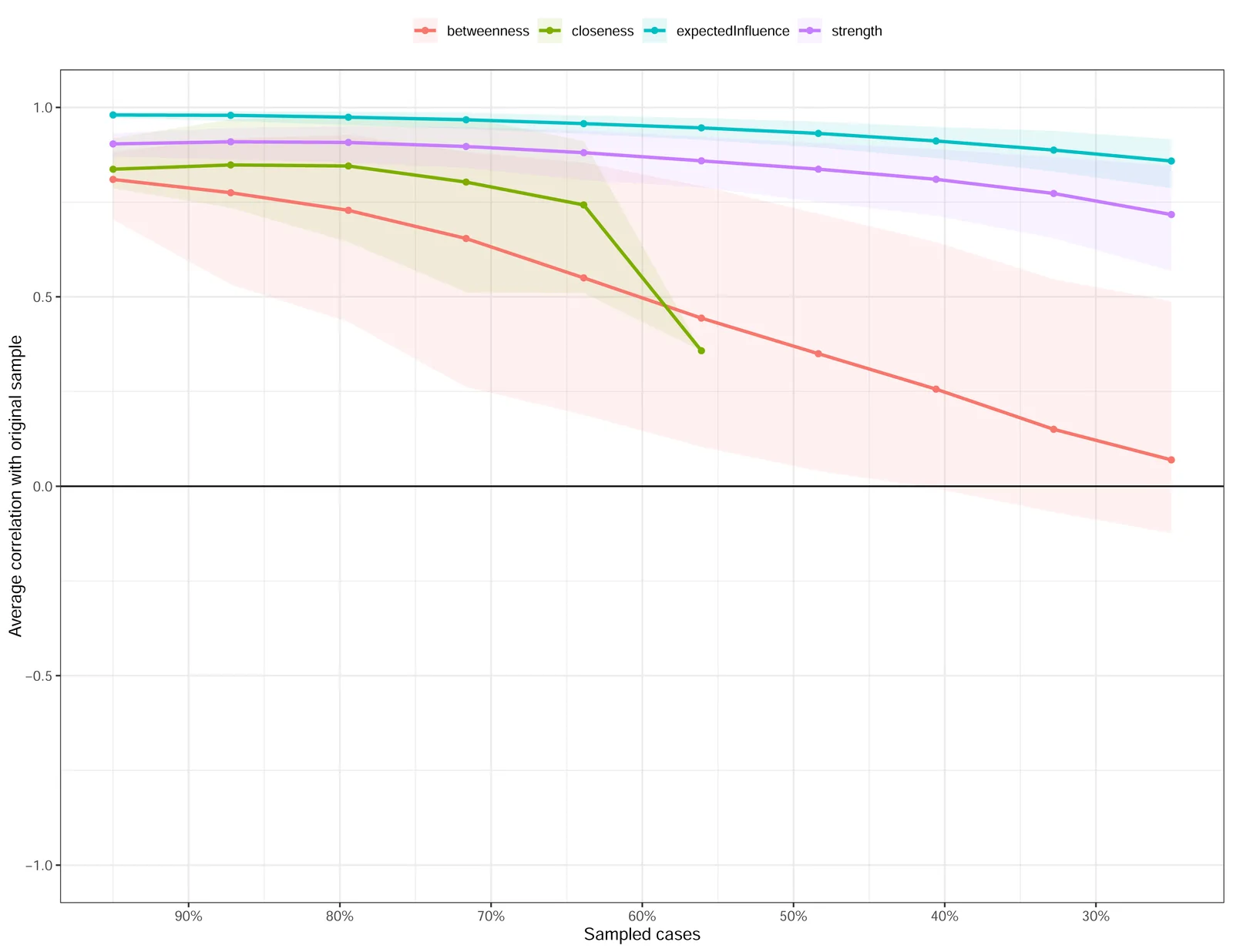
Accuracy of centrality measures. *X* axis represents the percentage of the original sample size that is maintained in case-dropping bootstrapped samples, whereas *Y* axis represents the correlation of the centrality measures between bootstrapped samples values and the original sample values. Colored areas represent confidence intervals. Closeness measures were highly unstable, so that they could not be calculated beyond a certain point. Betweenness showed poor accuracy as well, having a correlation of around 0.50 when 60% of the sample size was maintained and going close to null correlation in 25% of the sample size. Strength had acceptable accuracy, having more than 0.50 correlation with the original sample at 25% of the sample size. However, expected influence showed the greatest accuracy, keeping more than 0.75 correlation at 25% of the sample size.

### Main results

Network analysis results are plotted in Fig. 3. A network was generated with 58 non-zero edges from 528 possible edges (density = 10.98%).

**Figure 3:**
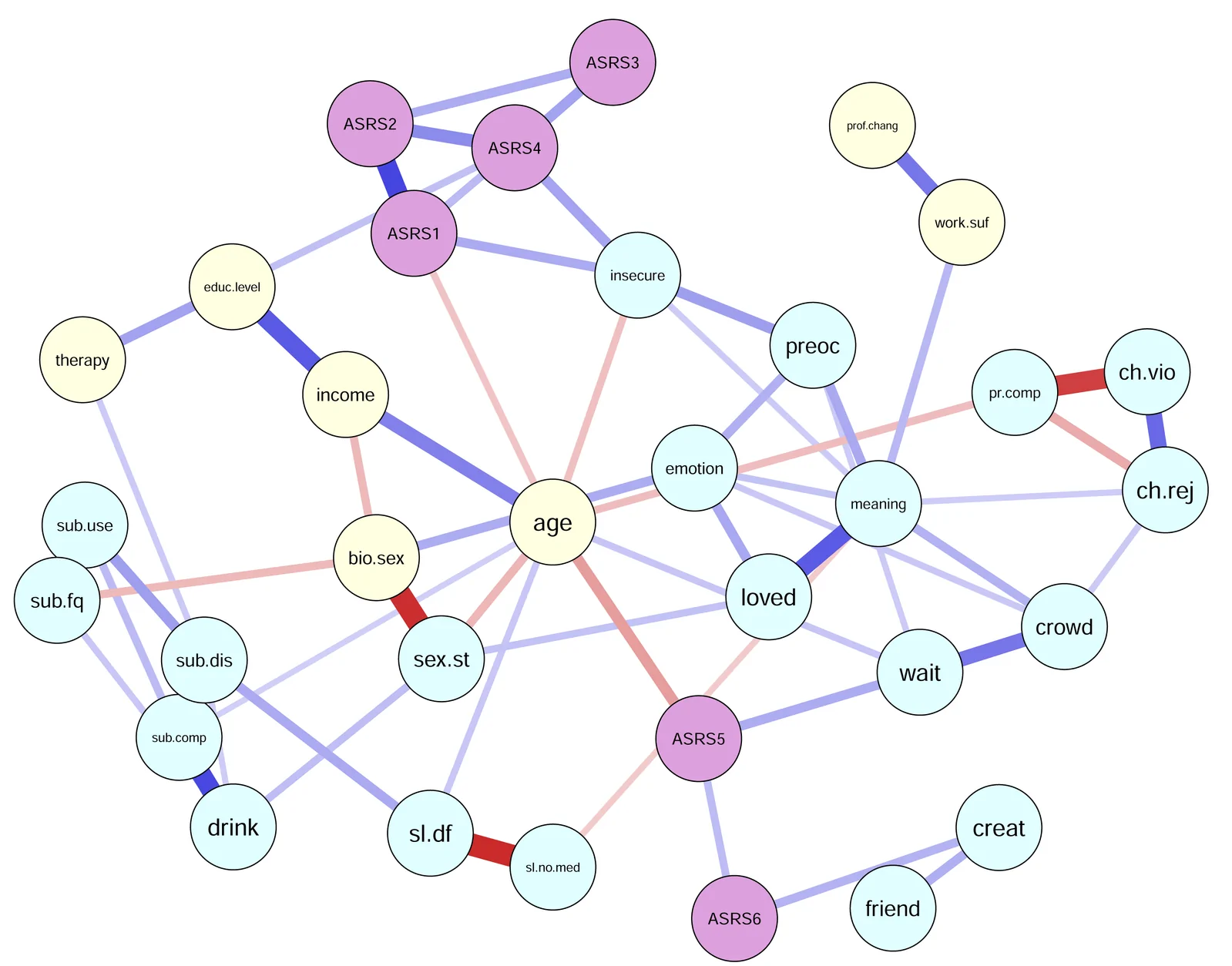
Network analysis main results. Circles represent nodes. Blue lines represent positive correlations, whereas red lines represent negative correlations. Line thickness represents the magnitude of correlation. All nodes had edges with at least one other node. ADHD traits are isolated above (inattention) and below (hyperactivity/impulsivity). See Table 1 for node labels. ADHD traits are highlighted in magenta, sociodemographic variables in yellow and mental health indicators in cyan.

ADHD inattention traits formed a cluster, having the trait of disorganization as the most connected among the four of them. The edge of highest magnitude between inattention traits was the edge between difficulty doing tasks and disorganization. Procrastination and difficulty doing tasks bridged the relationship between inattention traits and nodes associated with poorer mental health through their edges with insecurity doing tasks, which was the only mental health indicator directly associated with inattention traits. Procrastination was more prevalent in more educated individuals. Forgetting appointments and disorganization were relatively isolated inattention traits, having no edges outside of the inattention cluster.

Hyperactivity/impulsivity traits had an edge between themselves and no edge with inattention traits, which suggests a partial dissociation between these dimensions. Restlessness was the least connected ADHD trait, however serving as a bridge to positive traits like creativity and ease of making new friends. Psychomotor agitation was correlated with lower age and more difficulty waiting in lines, which can be interpreted as a logical consequence of that trait.

No node was isolated without an edge connected to itself. However, ADHD traits assumed a rather peripheral place in the network. No ADHD trait was directly linked to strong indicators of distress such as excessive preoccupation, difficulty sleeping, occupational suffering and lack of meaning in life. No node related to substance use, which is commonly associated with ADHD, was connected to any ADHD trait.

As for the rest of the network, it should be noted that biological sex was coded as 0 = male and 1 = female; so the node for sex represents the effect of being female, rather than male. Even controlling for the effect of all other variables, like age, education and mental health indicators, women declared lower income. Sex had no direct edge with ADHD traits. However, younger individuals declared more difficulty and insecurity doing tasks.

Centrality measures are presented in Fig. 4. Age was the most connected and, therefore, the most central node to the network in terms of strength. Lack of meaning in life was highly central both in expected influence and strength, as it was linked to multiple nodes associated with poorer mental health, but it had no edge with ADHD traits. The most central ADHD trait in the network was procrastination, as it was connected to other inattention traits, education and insecurity doing tasks.

**Figure 4:**
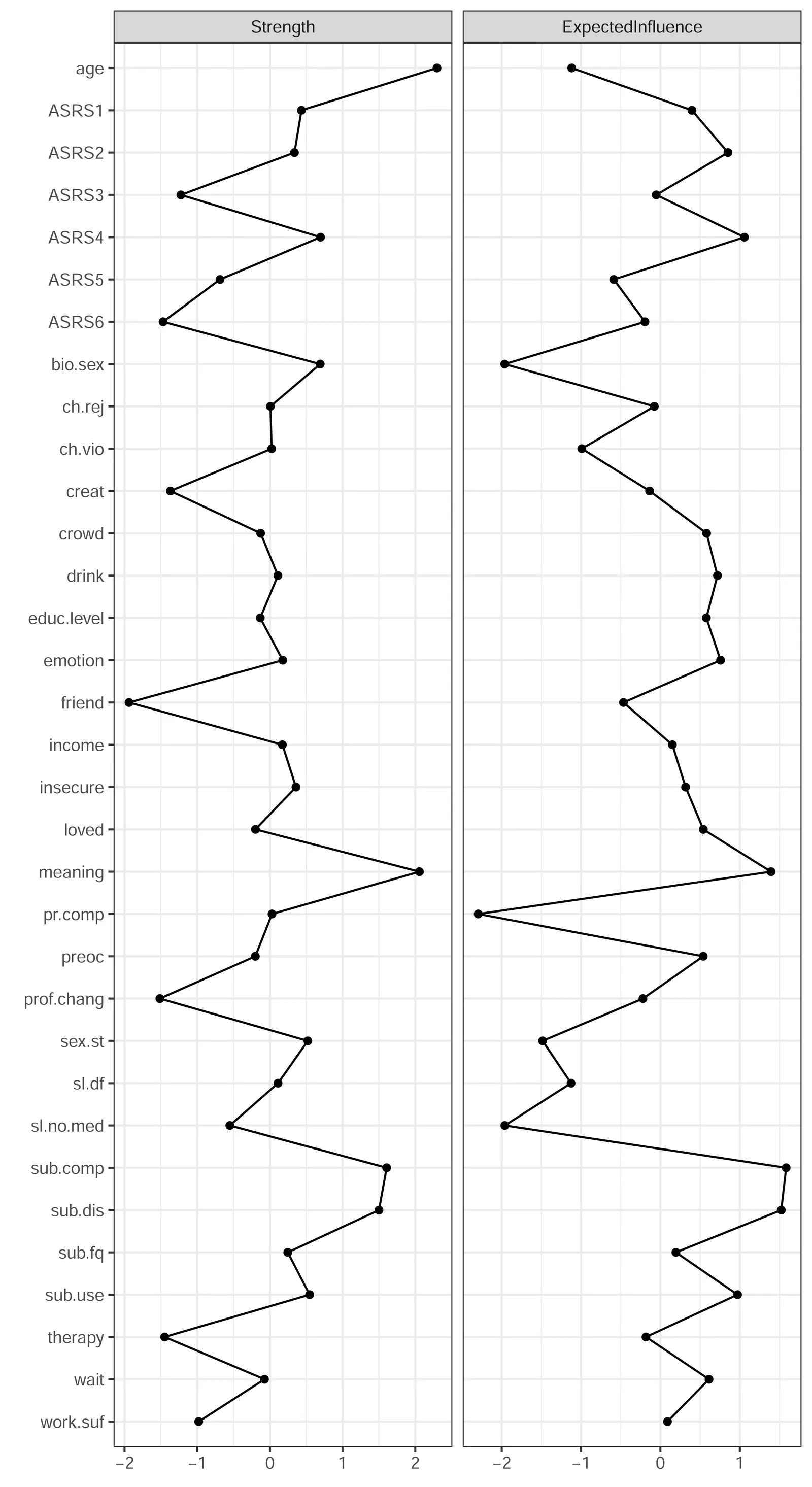
Centrality measures. Strength (left panel) and expected influence (right panel) measures are shown for every node (*Y* axis). In *X* axis, values are represented as mean (equal to 0) and standard deviation. Age and meaning have the highest strength. Meaning is also among the nodes with highest expected influence. Age’s expected influence is lowered once it has multiple positive and negative edges connected to it. Substance use nodes have high strength and expected influence due to the highly connected cluster they form. ASRS4 has both the highest strength and expected influence among ASRS items. See Table 1 for node labels.

### Redundancy analysis

UVA found eight redundant pairs of nodes: sub.use/sub.fq (wTO = 0.398), sub.comp/sub.dis (wTO = 0.333), sl.no.med/sl.df (wTO = 0.329), prof.chang/work.suf (wTO = 0.318), drink/sub.comp (wTO = 0.296), ch.vio/pr.comp (wTO = 0.265), ASRS1/ASRS2 (wTO = 0.262), and bio.sex/sex.st (wTO = 0.261). Network analysis was then applied to the reduced dataset in which each group of redundant nodes were reduced to their mean. Although biological sex (bio.sex) and excessive need of sexual stimuli (sex.st) were considered redundant by UVA (women scored lower on this item), these nodes were not reduced to their mean, as they represent two distinct pieces of information.

The main results were mostly replicated in the reduced network (Figs. S5 and S6). However, a low magnitude edge appeared between the node that represents the mean of ASRS1 and ASRS2 (Difficulty doing tasks and Disorganization) and the node that represents the mean of the items related to difficulty sleeping.

### Exploratory analyses

From the 2,159 individuals in the sample, 843 (39.05%) reported having never had any struggle regarding mental health. We performed a network analysis filtering the sample only for those individuals in order to explore if a history of mental health conditions may have affected our main results. Figure 5 displays the network analysis graph for this subsample. A sparser network was generated due to sample size limitation. ASRS 6 (Restlessness) became isolated, as did making new friends, creativity and attendance to therapy. As edges connected to those nodes were all of low magnitude in the full sample network, this outcome was partially expected. In all other respects, our main findings regarding other ADHD traits were replicated in this subsample. Therefore, we decided to test other hypotheses regarding these findings.

**Figure 5:**
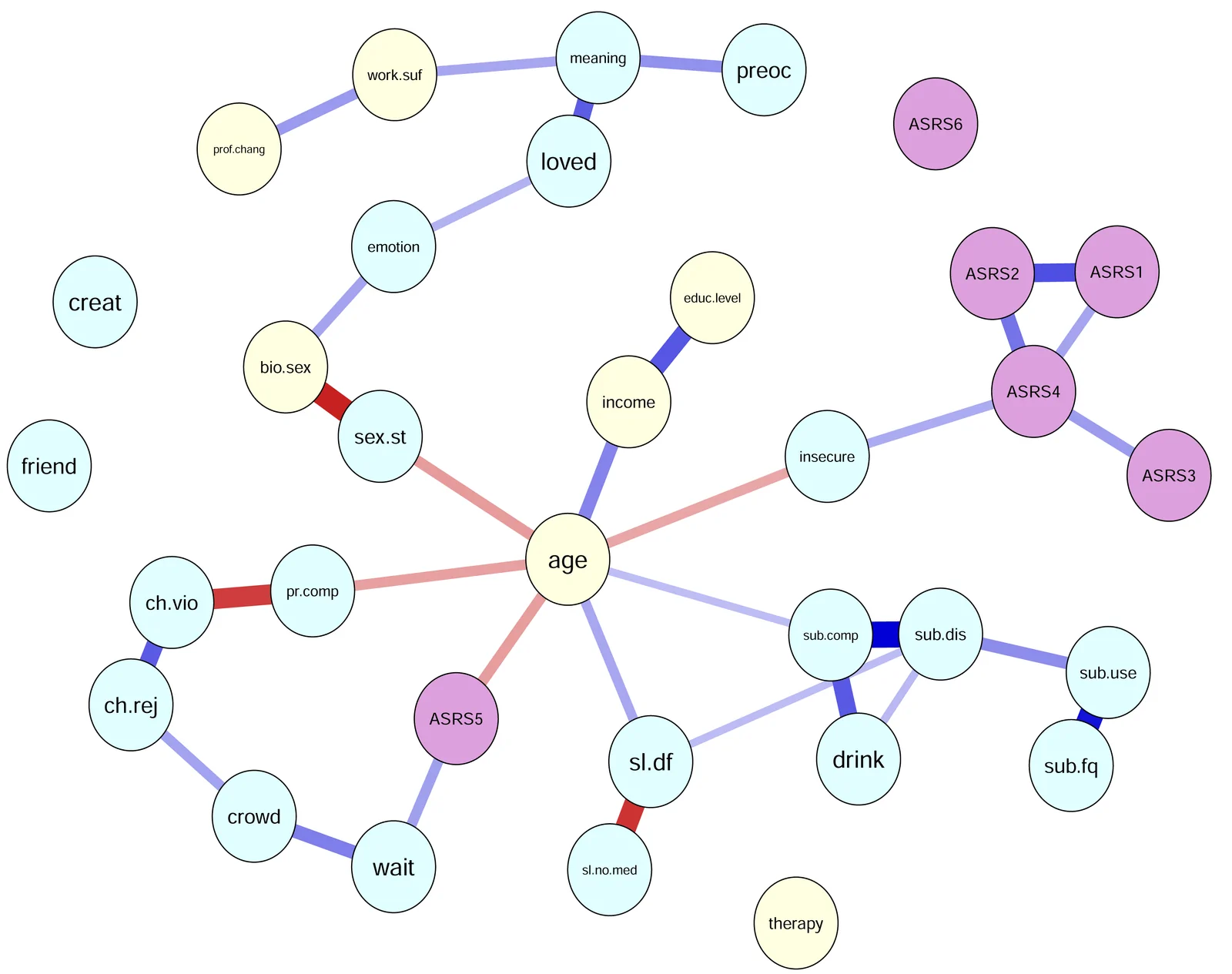
Network for subsample with no history of mental health struggles. Some low magnitude edges are no longer present, but most edges related to the main findings are replicated. See Table 1 for node labels. ADHD traits are highlighted in magenta, sociodemographic variables in yellow and mental health indicators in cyan.

It might be possible that no isolated trait has enough impact on mental health indicators for a link between them to be observed, but the summed effect of those traits might have a relevant impact. Thus, two other network analyses were employed to assess this possibility: in one network, all six ASRS items were summed into one node; in the other, the four inattention items were summed into one inattention node, and the same was done to the two hyperactivity/impulsivity items.

Both networks are presented in Fig. 6. ASRS full score had edges with insecurity doing tasks, difficulty waiting in lines, and age, all previously observed in the analysis of individual traits, but a new low magnitude edge with excessive need for sexual stimuli appeared. The inattention score had edges with insecurity doing tasks and age, while hyperactivity/impulsivity score had edges with age, difficulty waiting in lines, and a new edge emerged between hyperactivity/impulsivity and getting emotional frequently. However, this was an edge with low magnitude and accuracy.

**Figure 6:**
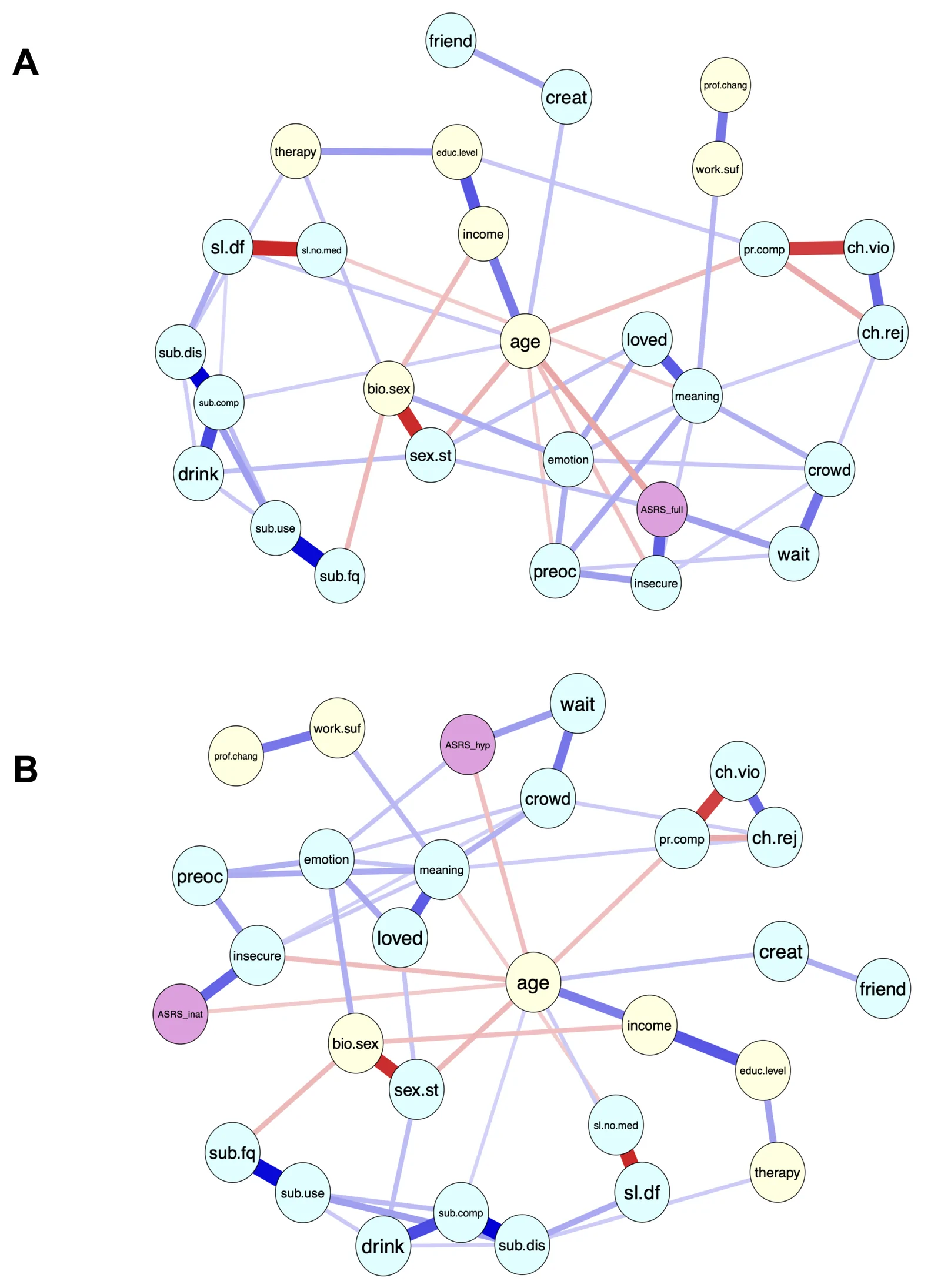
Networks with ASRS full score or inattention and hyperactivity/impulsivity scores. (A) Network with a node for ASRS full score (ASRS_full). (B) Network with a node for inattention score (ASRS_inat) and another one for hyperactivity/impulsivity score (ASRS_hyp). ASRS full score had edges with insecurity doing tasks (insecure), difficulty waiting in lines (wait) and age. Inattention score had edges with insecurity doing tasks (insecure) and age, while hyperactivity/impulsivity score had edges with age and getting emotional frequently (emotion). See Table 1 for node labels. ASRS summed scores are highlighted in magenta, sociodemographic variables in yellow and mental health indicators in cyan.

Another possibility would be that, although ADHD traits were not directly linked to most mental health indicators when the full range of trait presentation is considered, maybe these links are only present in highly inattentive, hyperactive and impulsive individuals. To assess this possibility, network analysis was also applied to two subgroups of the sample. One subgroup was composed of the 482 participants who were positive screens in ASRS (≥14). The other subgroup was composed of 713 individuals with ASRS scores equal or lower than 8, an arbitrary number lower than the sample mean (= 10.45) selected to maximize sample size while also creating a large gap of trait presentation between the two groups. Once sample size was significantly lowered, EBIC tuning parameter was reset to 0.25 (Hevey, 2018) to minimize edges discarded due to low sample size power (at the cost of higher risk of type I error).

Both are presented in Fig. 7. Two highly sparse networks were generated. Although a few significant edges were estimated, most of them are maintained in both networks, indicating similar behavior of the variables in the groups. Despite sample size limitations, which were mitigated by tuning parameter adjustment, results do not support that in individuals with high trait presentation the links between ADHD traits and mental health indicators are intensified.

**Figure 7:**
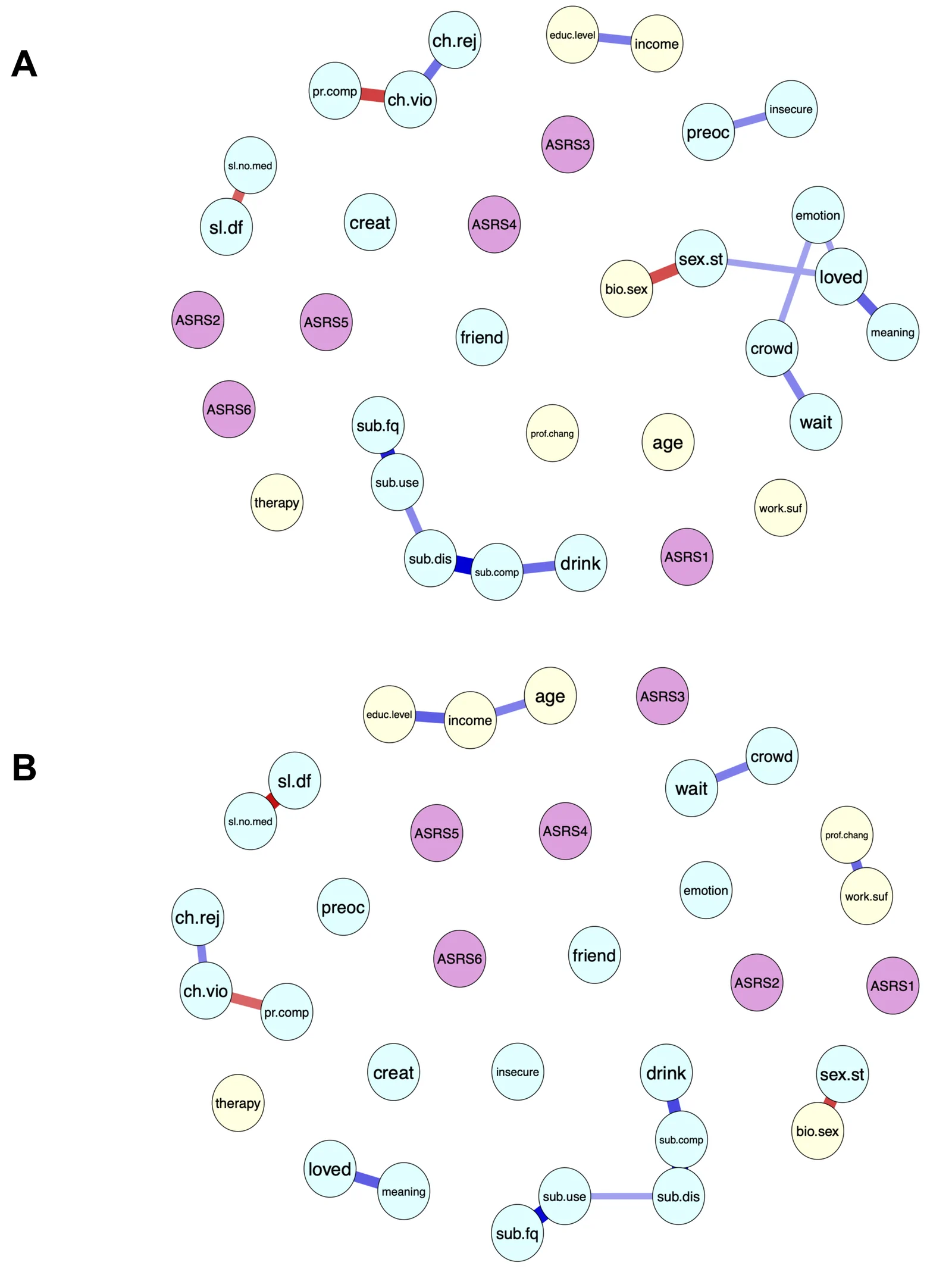
Networks for low ASRS score or positive screening participants. (A) Network for low ASRS score participants (≤8). (B) Network for positive screening participants (≥14). In both networks, substance use and childhood-related nodes formed clusters. The edge between education and income is present in both networks. In the positive screening network, feeling life has no meaning (meaning) and feeling excessive need to be loved (loved) had one edge between each other, and feeling distress in crowded places (crowd) and difficulty waiting in lines (wait) had another edge between each other, while in the low score network those four nodes formed a larger cluster together with excessive need of sex stimuli (sex.st) and biological sex. No edge was connected to an ADHD trait. See Table 1 for node labels. ADHD traits are highlighted in magenta, sociodemographic variables in yellow and mental health indicators in cyan.

### Comparison to bivariate correlations

Network analysis works with partial correlations, which adjust the correlation between two variables to their correlations with all other variables in the dataset. We compared network analysis results with what would be found should only bivariate correlations be calculated to explore how our findings would change when we do not take into consideration all correlations within the dataset. This could help to understand differences between our findings and those from correlational studies that did not employ network analysis. Table 3 displays results for Spearman’s correlation between ASRS full score and mental health indicators with associated *p*-values adjusted with Benjamini–Hochberg False Discovery Rate (FDR) correction for multiple testing.

**Table 3:** Bivariate correlations with ASRS full score.

**Variable**	**Spearman’s *ρ* correlation**	***p*-value (FDR)**
insecure	0.479	¡0.001
meaning	0.373	¡0.001
preoc	0.356	¡0.001
wait	0.326	¡0.001
emotion	0.315	¡0.001
loved	0.309	¡0.001
ch.rej	0.301	¡0.001
crowd	0.294	¡0.001
sex.st	0.267	¡0.001
sl.df	0.254	¡0.001
sub.dis	0.246	¡0.001
ch.vio	0.195	¡0.001
sub.comp	0.194	¡0.001
sub.use	0.190	¡0.001
sub.fq	0.171	¡0.001
drink	0.166	¡0.001
friend	−0.004	0.8612
creat	−0.153	¡0.001
sl.no.med	−0.157	¡0.001
pr.comp	−0.165	¡0.001

Only one variable (ease of making new friends) did not have a significant correlation with ASRS full score. Some correlations were as high as 0.373 for feeling life has no meaning, 0.356 for excessive preoccupation, and 0.301 for experiencing rejection in childhood, none of which had any significant direct correlation with ASRS full score or ADHD individual traits in network analysis, which implies that the bivariate correlation between those variables is actually bridged by other variables. On the other hand, insecurity doing tasks was pointed by network analysis as a possible bridge between ADHD traits and other mental health indicators, and it was also the variable with highest bivariate correlation with ASRS full score (*ρ* = 0.479).

## Discussion

Network analysis in mental health is a growing but relatively new field. In this study, we aimed to use network analysis in order to explore how ADHD traits are associated with mental health in the general population, independently of meeting the full diagnostic criteria. In doing so, we expect to generate valuable insight for future research in the field. In the following section, we discuss the insights brought by our findings.

Regarding sociodemographic variables, our results suggest a negative correlation between age and ADHD traits, especially with hyperactivity/impulsivity. Moreover, age was a highly central node in the main results network. ADHD is in fact more commonly observed in younger individuals, especially those between 18 and 29 years old (Adler et al., 2019). Those findings reinforce the profound impact of age differences in adult ADHD traits. Why do younger individuals report more ADHD trait presentation is a matter yet to be further understood, some possible hypotheses for this finding being that ADHD traits are diminished in the process of ageing (Polanczyk et al., 2010), both as a consequence of ageing-associated processes (feeling less energized) or the development of coping skills, or that ADHD traits are becoming more prevalent in younger populations due to societal factors.

Despite evidence showing that ADHD adults have worse occupational outcomes (Chen et al., 2024), no direct link was observed between ADHD traits and labor-related variables, such as experiencing more suffering in the workplace. Also, in contrast to evidence indicating that ADHD adults have decreased earning and higher rates of unemployment (Fletcher, 2014), ADHD traits had no direct link with income. However, a weak edge indicated that ADHD traits are associated with higher educational levels. What processes are influencing the association between ADHD traits and poorer outcomes is a compelling question which is a central corollary of our findings. Although the association between ADHD and poorer occupational and educational outcomes is a largely replicated result in the literature (Chen et al., 2024), our findings do not support that these outcomes are directly associated with ADHD traits alone in the general population. This finding is in line with research that has been showing that ADHD traits seem to even flourish in some contexts (Ekman, Hiltunen & Gustafsson, 2021; Moore, McIntyre & Lanivich, 2021). Indeed, once functional impairment is necessary for ADHD diagnosis, poorer occupational and educational outcomes in diagnosed individuals should not come as a surprise. We hypothesize there are some putative factors that influence functional impairment in individuals with high ADHD traits presentation, leading some to underdevelop functional behavior and to meet criteria for diagnosis, while some other individuals are able to develop adequate functioning. Social support throughout development (Richards, 2013) and in the workplace (Sedgwick, Merwood & Asherson, 2019) are hypothesized to have significant influence in that regard.

Our findings suggest that, in an adult community sample, ADHD traits have few significant direct correlations with mental health indicators. Most of these correlations are instead bridged by other variables, such as age and insecurity. Although rather atypical results, even in exploring other approaches to the analysis, such as analyzing subgroups of extreme trait presentation and summing ASRS scores, our general findings were unaltered. This indicates that direct correlation between ADHD traits and mental health indicators is not observed even in individuals with high trait presentation and is not affected by the summed effect of traits taken together.

However, some insight regarding possible associations between ADHD traits and mental health indicators can be derived from our findings. The topology of the main results network indicated that insecurity serves as a bridge between ADHD inattention traits and nodes highly associated with worse mental health, like excessive preoccupation. Therefore, we propose that the interaction between ADHD traits and insecurity is highly impactful and should be further investigated in future research. Although insecurity is not determinant of poor mental health *per se*, it can be a risk factor (low self-efficacy). We hypothesize that mental health treatments addressing insecurity could weaken the association between ADHD traits and worse mental health outcomes.

Another indicator consistently associated with ADHD traits was difficulty waiting in lines. Although it might be considered a part of ADHD traits itself, it can also be a sign of factors that impair functioning (*e.g.*, impatience). Getting emotional frequently was weakly associated with hyperactivity/impulsivity as well. Although it was a finding of low accuracy, when taking into account that insecurity was the mental health indicator most associated with ADHD traits, this scenario builds a case for emotional factors as important variables to be included in ADHD research. Previous research has already found associations between ADHD and higher emotion dysregulation (Gauld et al., 2025; Beheshti, Chavanon & Christiansen, 2020). Therefore, we also propose impatience and emotional factors as variables that may influence mental health outcomes associated with ADHD traits.

Network analysis showed that restlessness was positively correlated with healthy traits, like creativity. This finding is in line with previous observation that positive characteristics are more commonly associated with hyperactivity than with inattention (Sônego et al., 2021). Although quite modest, once the observed edges were relatively weak, we consider this finding to be highly relevant. Previous research has shown that positive characteristics are reported to be associated with ADHD, especially creativity (Schippers et al., 2022). In fact, most individuals report that ADHD has positive characteristics associated with it (Mahdi et al., 2017). This builds up evidence for a less inherently pathological and more context-dependent view of ADHD traits outcomes (Ekman, Hiltunen & Gustafsson, 2021; Moore, McIntyre & Lanivich, 2021). Positive outcomes associated with ADHD are not commonly assessed in present research but are a promising theme for future studies. In that regard, we would consider it especially relevant to investigate how the interaction between ADHD traits and social factors influences the outcomes that these traits will produce.

Comparison of network analysis results to bivariate correlation results is intriguing. ADHD is highly associated with multiple mental health impairments (Lugo et al., 2020), and this finding would be replicated in our data through bivariate correlation. However, through network analysis using partial correlations, we found that most correlations between ADHD traits and mental health indicators are actually bridged by other variables. This finding imposes a great challenge to research, as it suggests that some studies, findings should be replicated using data analysis methods that consider possible confounding effects in the associations that have been found between ADHD traits and mental health impairment.

These findings must be interpreted considering their limitations. The number of individuals who were positive screens in ASRS (22.33% of the sample) exceeded previous estimations for the Brazilian population. In a 2010 study (Polanczyk et al., 2010), a representative household sample of 3,007 Brazilian individuals was screened for ADHD using the ASRS, which resulted in an estimate of 5.80% (95% CI [4.80–7.00]). In a more recent study of 2024, the ASRS was used to estimate ADHD positive screens in the city of Rio de Janeiro, Brazil, which might be used as a proxy for the Brazilian population of today. In the stratified and clustered probability sample of 2,297 individuals, 4.59% (95% CI [3.56–5.44]) were positive screens (Mattos et al., 2024). The elevated prevalence of positive screens in our sample could be attributed to the survey title, which may have biased the sample towards individuals with an interest in mental health, although it is not possible to guarantee if the subjects had ADHD or any mental health diagnoses. Despite that, this design enabled the recruitment of a large sample of participants.

A major limitation of this study was the use of items made on-demand instead of already validated instruments to assess mental health. We sought to overcome this limitation by presenting evidence of validity for these items, which demonstrated adequate validity. However, future studies will benefit from using validated instruments for mental health assessment, as we may have missed important indicators with our items. We also had the limitation of using only ASRS part A items. Although these items are the best predictors of ADHD positive screening, we think future studies may expand and deepen our findings by using more complete instruments, as possible links between mental health and ADHD traits may have been overshadowed by underrepresentation of ADHD traits.

Our sample is limited by the elevated prevalence of highly educated individuals, which can make our findings somewhat restricted to this group. Therefore, we would consider it profoundly relevant for future research to try and replicate these findings in less educated samples. Our findings may also reflect some aspects that are specific to the Brazilian population and culture. However, rather than a limitation, our sample from a population underrepresented in mental health studies brings new insights into mental health research and contribute to the transcultural replication of research findings.

## Conclusions

Mental health in general and ADHD in particular are complex phenomena. Therefore, dealing with their complexity is necessary for their proper understanding. Network analysis was found to be a useful tool and appropriate theoretical background for this end.

Our findings suggest that ADHD traits presentation is not by itself determinant of mental health impairment in the general population; rather, some other factors, such as younger age and feelings of insecurity, have intervening effects that establish or strengthen the association between ADHD traits and mental health impairment. We expect that future research will be able to identify other factors that may have the same effect. We also hope that future research benefits from the insights derived from our study.

##  Supplemental Information

10.7717/peerj.21802/supp-1 Figure S1 Exploratory factor analysis results for mental health indicators

10.7717/peerj.21802/supp-2 Figure S2 Factor loadings for mental health indicators items

10.7717/peerj.21802/supp-3 Figure S3 Exploratory factor analysis results for ASRS items

10.7717/peerj.21802/supp-4 Figure S4 Factor loadings for ASRS items

10.7717/peerj.21802/supp-5 Figure S5 Network analysis with reduced dataset from redundancy analysis Circles represent nodes. Blue lines represent positive correlations, whereas red lines represent negative correlations. Line thickness represents the magnitude of correlation. See Table 1 for node labels. ADHD traits are highlighted in magenta, sociodemographic variables in yellow, mental health indicators in cyan and nodes that represent the mean of redudant nodes are in pink.

10.7717/peerj.21802/supp-6 Figure S6 Centrality measures with reduced dataset from redundancy analysis Strength (left panel) and expected influence (right panel) measures are shown for every node (*Y* axis). In *X* axis, values are represented as mean (equal to 0) and standard deviation. See Table 1 for node labels.

10.7717/peerj.21802/supp-7 Supplemental Information 7 R Code

10.7717/peerj.21802/supp-8 Supplemental Information 8 ADHD dataset

## Additional Information and Declarations

### Data Availability

The following information was supplied regarding data availability: The data and code are available in the Supplemental Files. The Labor stressors influencing disability in Adult Attention Deficit/Hyperactivity dataset is available at Mendeley: ABRAMOV, DIMITRI (2020), “Labor stressors influencing disability in Adult Attention Deficit/Hyperactivity”, Mendeley Data, V1, doi: 10.17632/s9sdxddnnx.1
