# Supplementary material for: Network analysis of attention deficit hyperactivity disorder traits and mental health indicators in Brazilian adults
Source: PeerJ. 2026 Nov 3;14:e21802. doi: 10.7717/peerj.21802 (PMC13741977; doi:10.7717/peerj.21802)
Supplement: Supplementary file 1 [file peerj-14-21802-s005.pdf]

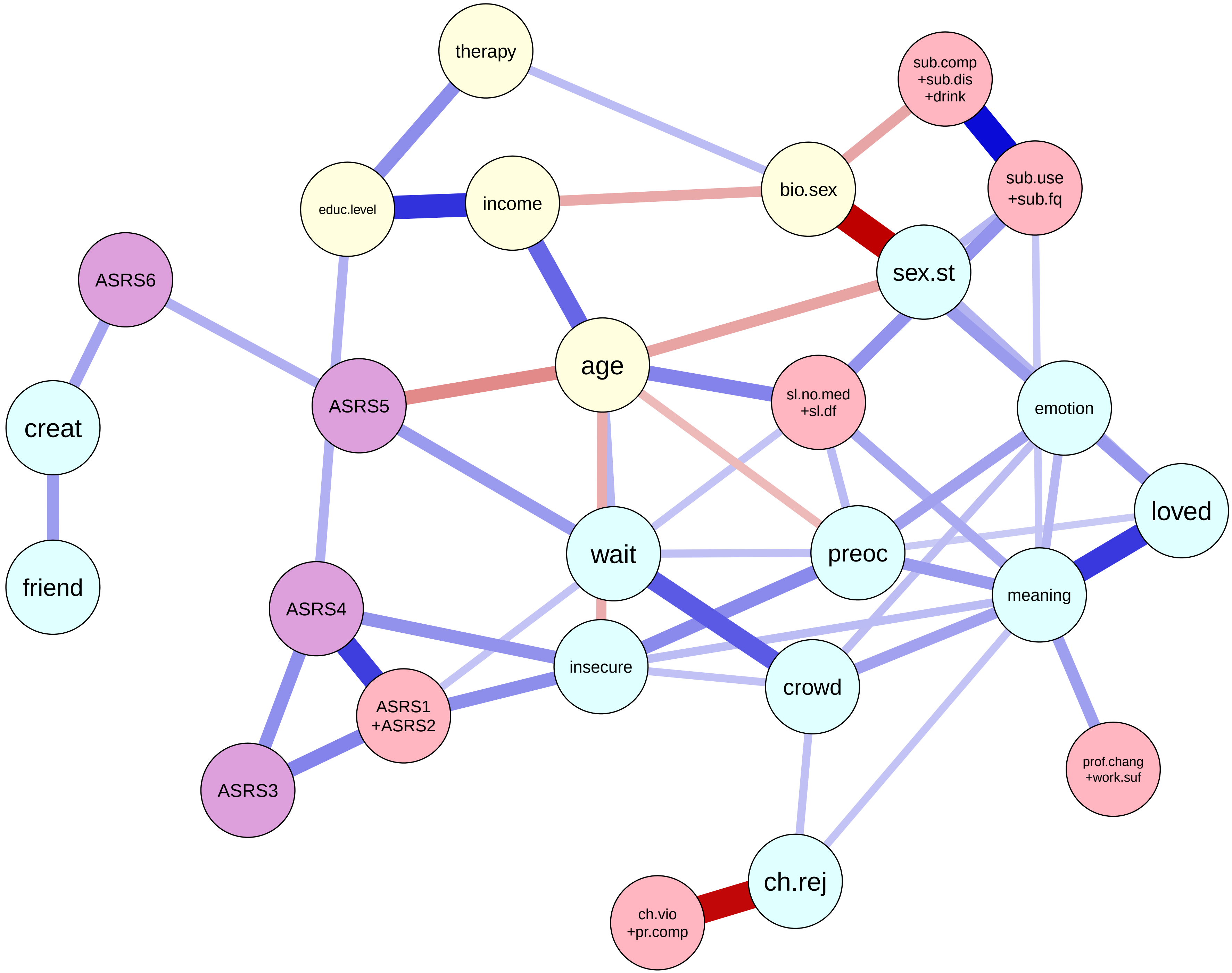
therapy
sub.comp
+sub.dis
+drink
educ.level
income
bio.sex
sub.use
+sub.fq
ASRS6
sex.st
age
creat
ASRS5
sl.no.med
+sl.df
emotion
loved
wait
preoc
friend
meaning
ASRS4
insecure
ASRS1
+ASRS2
crowd
prof.chang
+work.suf
ASRS3
ch.rej
ch.vio
+pr.comp
