# Supplementary material for: Network analysis of attention deficit hyperactivity disorder traits and mental health indicators in Brazilian adults
Source: PeerJ. 2026 Nov 3;14:e21802. doi: 10.7717/peerj.21802 (PMC13741977; doi:10.7717/peerj.21802)
Supplement: Supplementary file 2 [file peerj-14-21802-s006.pdf]

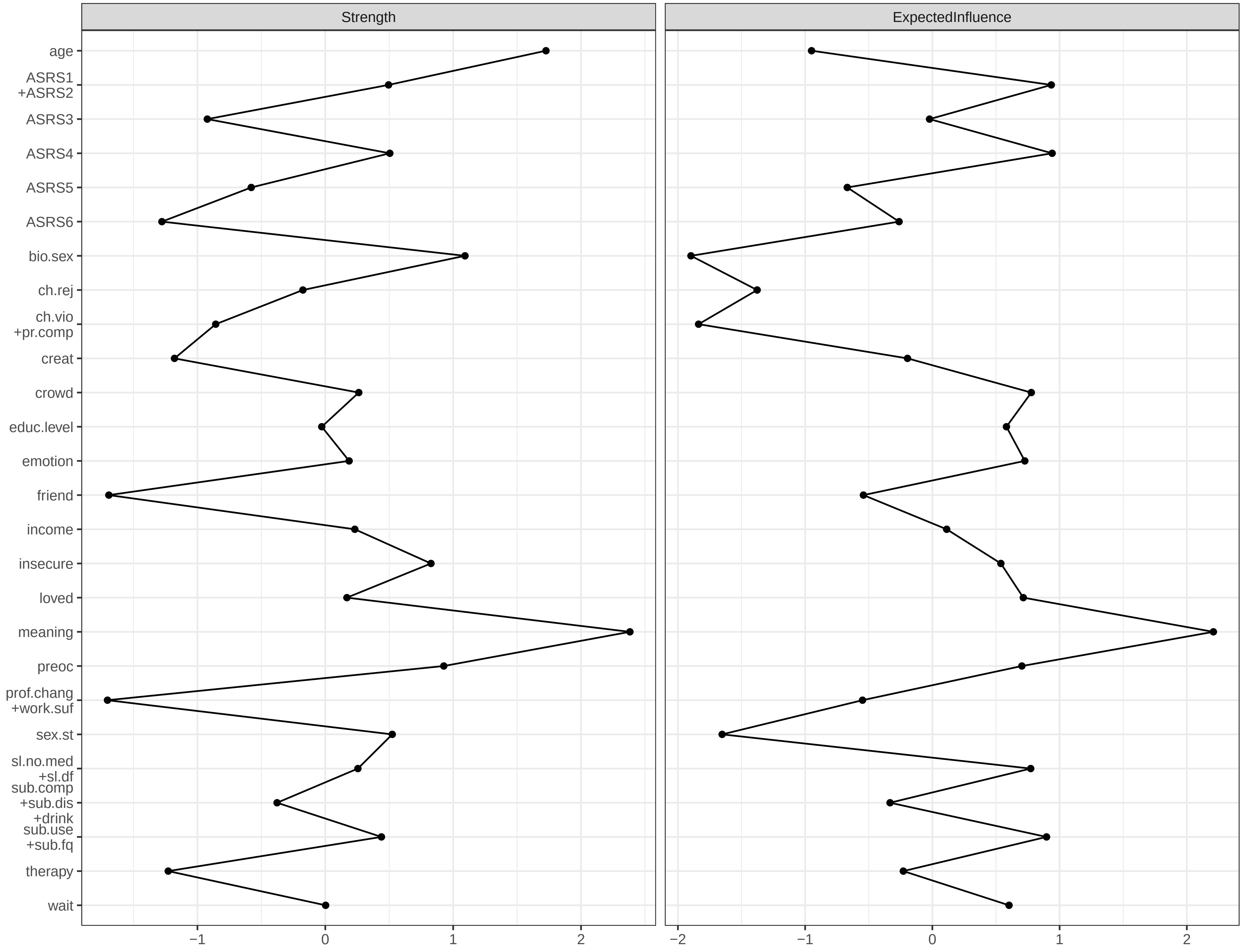

Strength
ExpectedInfluence
age
ASRS1 +ASRS2
ASRS3
ASRS4
ASRS5
ASRS6
bio.sex
ch.rej
ch.vio +pr.comp
creat
crowd
educ.level
emotion
friend
income
insecure
loved
meaning
preoc
prof.chang +work.suf
sex.st
sl.no.med +sl.df
sub.comp +sub.dis +drink
sub.use +sub.fq
therapy
wait
−1
0
1
2
−2
−1
0
1
2
